# Developmental Variation of Indian Thermophilic Variety of Scuttle Fly* Megaselia (Megaselia) scalaris* (Loew, 1866) (Diptera: Phoridae) on Different Substrates

**DOI:** 10.1155/2016/4257081

**Published:** 2016-07-04

**Authors:** Abesh Chakraborty, Atanu Naskar, Panchanan Parui, Dhriti Banerjee

**Affiliations:** Zoological Survey of India, Ministry of Environment & Forests (Government of India), M Block, New Alipore, Kolkata 700 053, India

## Abstract

The scuttle flies (Diptera: Phoridae) are important in forensic dipterology, because of their necrophagous habit. They are amongst the first wave of insects visiting human corpses in mechanically barricaded environments; hence their immature stages are generally used for estimation of PMI. The effect of different substrates commonly used for developmental studies was studied to analyze the variation of growth of the thermophilic variety of* Megaselia (M.) scalaris *prevalent in India on GDM, EDM, and SMS (*n* = 3). One approach of PMI estimation depends on larvae collected from the crime scene and comparing them with reference data derived from larval rearing to establish PMI. Results showed that there was a significant variation in avg. length (*F*(2,111) = 15.79873, *p* = 0.000000917), width (*F*(2,111) = 14.60528, *p* = 0.00000234), and biomass (*F*(2,111) = 37.01727, *p* = 0.000000000000482) of the immature stages in the three media and the larvae grow maximally in the SMS medium. The results of the present study thus provide baseline data on the growth and developmental pattern of the* Megaselia (M.) scalaris*, which can be utilized in conjunction with specific geoclimatic reference data, for forensic entomological studies and also for using the phorid as a biocontrol agent of pestiferous insects.

## 1. Introduction

The scuttle flies (Diptera: Phoridae) are among the late arrivals in the faunal succession wave on human and animal cadavers. These secondary colonizers of decomposing remains are sometimes primary colonizers, when the decomposing remains are mechanically barricaded or are placed in concealed environment. The adults together with the immature stages aid in both long and short term PMI estimation. The model organism used for the study is* Megaselia (Megaselia) scalaris* (Loew, 1866) which is a very cosmopolitan species. Recently, this species of scuttle fly has been utilized as a primary player in ecological research in studies focusing on the effect of physical and physiological conditions on the ethology and endogenous sensing abilities of the fly and for bio-control of pest insects [1–4]. The scuttle fly* Megaselia (Megaselia) scalaris* (Loew, 1866) has been found in tropical rain forest and urban jungle [5]. The immature stages of this species have been described as detritivore, parasite, facultative parasite, and parasitoid, phytophagous, and coprophagous [5–7]. The adult is reported as polyphagous organism [8].

The parasitoid nature of the larvae of* M. M. scalaris* is most likely triggered by overcrowded conditions. Field reports have demonstrated the ability of this fly to feed on a huge range of living arthropods, including members of the following orders: Orthoptera [9], Diptera [10], Lepidoptera [11, 12], Coleoptera [13, 14], Hymenoptera [15], Ixodida [16], and Araneae [17], some of which are of agro economic and ecological importance. Its extraordinary ecological plasticity has also led to the establishment of* M. M. scalaris* as a lab pest, having known to infest laboratory cultures of invertebrates such as cockroaches [18, 19], flies [20], and triatomines [8]. Therefore, understanding their life cycle and development pattern in different substrate is essential for using* M. M. scalaris* as a biocontrol agent for ecological, agronomic studies and also forensic entomological studies. The scuttle fly* M. M. scalaris* (Loew) is a forensic dipteran and is useful in estimating postmortem interval for humans, time since death for animals, and time of negligence for both humans and animals. Here we present the effect of different diets on rearing of this fly, by feeding it three different types of diets, namely, general* Drosophila* media (GDM), enhanced* Drosophila* media (EDM), and soy milk silicon (SMS) media, in laboratory conditions. In the present experiment, SMS media was the best one concerning growth and biomass of these flies.

Unfortunately, there is a lack of standardization of laboratory adult insects feeding, breeding, and rearing protocols among researchers in the fields of forensics, ethology, and ecology experiments. This paper is currently focused on basic culture requirements of* Megaselia (Megaselia) scalaris*, by comparing it with 3 diets, biometric study, and development rates of this fly. The adults and immature stages aid in forensics and ethological and ecological research [1, 2].

The current trends in research focus on* M. M. scalaris* as a model organism of interest across a variety of disciplines and therefore there is a need for streamlining breeding and culture conditions of these scuttle flies species. Mainx [21] is considered the father of modern scuttle fly culture, though he worked on the genetics of sex determination of* M. M. scalaris* (Diptera: Phoridae). Mainx's medium was further streamlined in Prawirodisastro and Benjamin, 1979 [2] and Mazyad and Soliman, 2006 [1]. These media generally focused on maximizing larval density in small space; unfortunately this also increased the pupations times and the duration of life stages. Therefore these diets were not addressing the needs of forensic dipterology, as alteration of time or development in pupa, egg, larva, and adult stage may cause hindrance in PMI calculations based on additional degree days (ADD) [2, 21].

In cultures of other flies of forensic and environmental forensic importance, laboratories have generally utilized such a recipe or its variants to make a semisynthetic medium for adult flies to decrease food source heterogeneity [22]. In an effort to standardize and simplify scuttle fly media, this paper compares length, width, and biomass in the 3 media to determine which diet provides optimum feeding, breeding, and culturing outcomes for* M. M. scalaris* for use in ecology (micro, decomposition, carcass, and behavioral), forensics (sentinels and indicators), and medicolegal estimations (postmortem interval and time of negligence).

The most common contribution of forensic dipterology is the establishment of postmortem interval (PMI) by collecting entomological evidences collected at the scene and culturing them to adulthood and acquiring lower threshold to calculate the unknown portion of the PMI [23–26]. Therefore medium and long range PMI can be estimated using the developmental, life cycle, and ecological data coupled with ADD calculation to find the lapse time; the principle can also be used in time of negligence (TON) studies of human and animal myiasis [24]. The secondary objective of the study is to generate the baseline data, namely, to aid estimation of ADD/ADH from data collected in controlled settings, and then to confirm them in field [27, 28].

MDT (maggot debridement therapy) colonies to clean the microenvironment, dead human, or animal tissue and controlled myiasis for wound healing can be done, if the particulars aspects of life cycle are known [29]. This therapeutic application is most beneficial to the treatment of diabetic and other necrotic ulcers, where festering tissue of patients poses health risk to patients [30, 31]. In such cases, larvae are applied to wound; larvae consume the dead tissue and excrete a saliva-like substance that contains antimicrobial properties; both functions aid in the therapeutic process [29]. MDT colonies are developed using similar methods to those used by forensic dipterologists [32].

The introduction of insect in genetics in forensic analysis greatly aggravated the importance of the methods and protocols used in laboratories to develop, establish, and reproducibly rear colonies from field collected species. It is essential for regularity to exist between laboratories performing experimenting on any type of molecular or microbial forensic analysis if specimens are to be analyzed and replicated in different labs. Besides, diversity of culture protocols has the potential to result in changes in morphometric and gene expression differences; thus there is a need for standardization of protocols [33]. There are a number of different processes for feeding, breeding, and rearing to develop blow fly cultures for forensic and medical entomology labs and similar process are also in place for* Drosophila* for genetic, ecological, and ethological research. But unfortunately there is a lack of such readily producible protocols for Phoridae culture for ecology (micro, decompositional, carcass, and behavioral), forensics (sentinels and indicators), and medicolegal estimations (postmortem interval and time of negligence).

To streamline the protocols and develop lab cultures, 3 media were tested for observing the efficacies of media on biometric study and development rates of phorids. The first was the general* Drosophila* medium, the second was enhanced* Drosophila* medium, and the third was SMS (soy milk SiO_2_) medium [1–21, 27, 34–45].

## 2. Materials and Methods

### 2.1. Source of Flies for Culture

Adult flies (*M. M. scalaris*, Bhavnagar strain collected in 2015) were taken for inoculating the stock cultures, courtesy of A. Naskar. Flies were reared in general* Drosophila* media till adult stage was reached, for the first generation; then the adults were placed in the three different media in small culture jars, namely, general* Drosophila* medium, enhanced* Drosophila* medium, and SMS (soy milk SiO_2_) medium, and were reared at constant temperatures of 27 ± 3°C, relative humidity of 35 ± 5%, and photoperiod of 12 : 12 (L : D) hrs.

### 2.2. Separation of Pupae and Initiating Culture

General* Drosophila* medium was prepared by (w/v) agar 0.625 gms, maize powder 6.25 gms, dried yeast 1.87 gms, and brown sugar 6.25 gms/water 75 mL. Enhanced* Drosophila* medium was prepared by (w/v) agar 0.625 gms, maize powder 6.25 gms, dried yeast 1 gms, dried mushrooms 0.5 gms, and brown sugar 6.25 gms/water 75 mL. SMS medium was prepared by (w/v) commercially available soy protein isolates 3.78 gms, milk protein concentrates 1.2 gms, and silicon dioxide 0.0125 gms/75 mL water. The medium was poured in autoclaved culture vials and cooled in laminar air flow [1–4, 21, 27].

### 2.3. Data Collection

The biometric study and development rates were measured and calculated; when one generation of adults completed their life cycle, they were collected by the usual taxonomic process. Biometric analysis was done under Light Microscope (Leica StereoZoom 40x) and development rates were calculated by observing and averaging individuals (*n* = 10) and notes were taken on a daily basis every hour, and data was prepared for every 12 hours, regarding the time taken by each stage in a particular medium.

### 2.4. Preservation

The adult phorids specimens were collected from culture and killed by ethyl ether and preserved in 70% ethyl alcohol vials of 5 mL. The larvae, pupae, and infested pupae were kept in 70% alcohol, after dipping them in 10% warm KOH sol. for 3 mins. Afterwards, a variant of Baber's solution was used for their preservation for further taxonomic studies and bio measurements.

### 2.5. Data Analysis

The biometric measurements were taken by Light Microscope (Leica StereoZoom 40x). The biometric study was calculated based on APHA series protocols. The raw data was subjected firstly to descriptive statistics (namely, mean, median, mode, max and min, variation, standard deviation, standard error, and confidence at 95% level) [34].

Afterwards the biometric data was graphed with the aid of scatter plot (ROD curve) and regression was done according to a modified process of Chen et al. [35].

Lastly ANOVA (1-way), followed by post hoc *t*-test Bonferroni corrected, was done and the means and variance were plotted in error bars to see the average variation levels according to Millar [36]. The effects of various diets (*n* = 3) on the growth and development of* M. M. scalaris* were studied.

## 3. Results and Discussion

The study was conducted in ZSI, Kolkata; latitude 22°30′51.6888′′ and longitude 88°19′30.5256′′ were recorded by GPS meter. The adult* M. M. scalaris* were inoculated in this medium, but they did not start reproducing immediately; mating was initiated around 24 ± 2 hours after inoculation of culture; ovipositing began 36 ± 2 hours after inoculation of the culture. In lab condition, it is found that if* M. M. scalaris* are reared at constant temperatures of 27 ± 3°C, relative humidity of 35 ± 5% and photoperiod of 12 : 12 (L : D) hrs in different media showed different growth patterns and biomass accumulations. The length and breadth of larvae of all the three instars, pupa, and eggs were measured using Leica EZ4 allied software.

### 3.1. Growth Rate Analysis

A one-way ANOVA was done between the three culture media's larval length (in mm), width (in mm), and biomass (in mm^3^), to compare the effect of different diets of variable composition on the growth patterns and trajectories of* M. M. scalaris*.

#### 3.1.1. Length

There was a significant effect on the amount of variation in length (in mm) of the three culture media at the *p* < 0.05 level for the three different cultures (*F*(2,111) = 15.79873, *p* = 0.000000917).

#### 3.1.2. Width

There was a significant effect on the amount of change in width (in mm) of the three culture media at the *p* < 0.05 level for the three different cultures (*F*(2,111) = 14.60528, *p* = 0.00000234).

#### 3.1.3. Biomass

**Figure 1:**
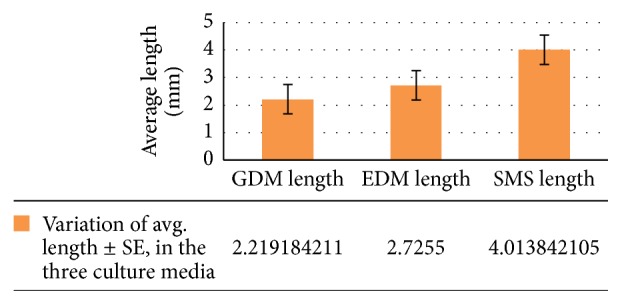
Variation of avg. length ± SE, in the three culture media.

**Figure 2:**
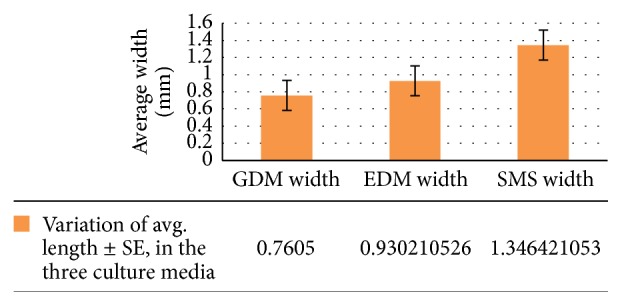
Variation of avg. width ± SE, in the three culture media.

**Figure 3:**
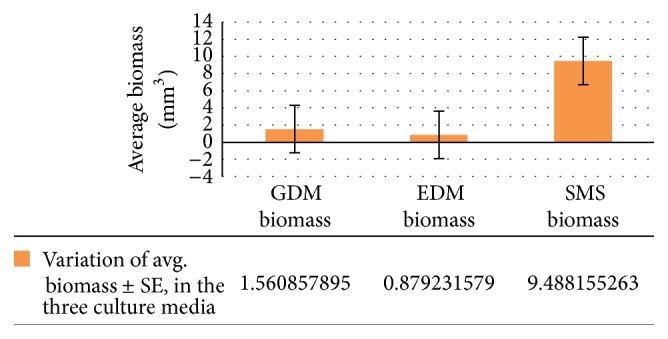
Variation of avg. biomass ± SE, in the three culture media.

There was a highly significant effect on the amount of change in biomass (in mm^3^) of the three culture media at the *p* < 0.05 level for the three different conditions (*F*(2,111) = 37.01727, *p* = 0.000000000000482) (see Figures 1, 2, and 3).

### 3.2. Statistical Analysis

Because we found a statistically significant result; therefore we need to compute a post hoc test. For this purpose we selected the *t*-test post hoc: two samples assuming equal variances and the significance levels were Bonferroni corrected. This test is designed to compare each of our immature stages' biometric data on different culture media to each other.

This test is generally designed to compare the GDM, EDM, and SMS culture media biometric data of the immature stages, in a three-way analysis, namely, length, width, and biomass (GDM and EDM, EDM and SMS, and GDM and SMS), respectively. The result of this post hoc* t*-test, two-tailed and assuming equal variances, Bonferroni corrected if significant, will affect the overall ANOVA.

#### 3.2.1. Length

For length (in mm) (GDM and EDM), *p*(*T* ≤ *t*) = 0.0412757681595545, Bonferroni correction for post hoc *t*-test (*α* = 0.05/3 = 0.0167); therefore GDM and EDM are not that significantly different from each other (0.0167 < *p*). EDM and SMS: *p*(*T* ≤ *t*) = 0.000741123157513785, Bonferroni correction for post hoc *t*-test (*α* = 0.05/3 = 0.0167); therefore GDM and EDM are significantly different from each other (0.0167 > *p*). GDM and SMS: *p*(*T* ≤ *t*) = 0.00000464959, Bonferroni correction for post hoc *t*-test (*α* = 0.05/3 = 0.0167); therefore GDM and EDM are significantly different from each other (0.0167 > *p*) (see Figure 1).

#### 3.2.2. Width

For width (in mm) (GDM and EDM), *p*(*T* ≤ *t*) = 0.0450198428605722, Bonferroni correction for post hoc *t*-test (*α* = 0.05/3 = 0.0167); therefore GDM and EDM are not that significantly different from each other (0.0167 < *p*). EDM and SMS: *p*(*T* ≤ *t*) = 0.00122566128758058, Bonferroni correction for post hoc *t*-test (*α* = 0.05/3 = 0.0167); therefore GDM and EDM are significantly different from each other (0.0167 > *p*). GDM and SMS: *p*(*T* ≤ *t*) = 0.00000902, Bonferroni correction for post hoc *t*-test (*α* = 0.05/3 = 0.0167); therefore GDM and EDM are significantly different from each other (0.0167 > *p*) (see Figure 2).

#### 3.2.3. Biomass

For biomass (in mm^3^) (GDM and EDM), *p*(*T* ≤ *t*) = 0.00197640275431817, Bonferroni correction for post hoc *t*-test (*α* = 0.05/3 = 0.0167); therefore GDM and EDM are significantly different from each other (0.0167 > *p*). EDM and SMS: *p*(*T* ≤ *t*) = 0.0000000137, Bonferroni correction for post hoc *t*-test (*α* = 0.05/3 = 0.0167); therefore GDM and EDM are significantly different from each other (0.0167 > *p*). GDM and SMS: *p*(*T* ≤ *t*) = 0.000000133783, Bonferroni correction for post hoc *t*-test (*α* = 0.05/3 = 0.0167); therefore GDM and EDM are significantly different from each other (0.0167 > *p*) (see Figure 3).

### 3.3. Developmental Pattern Analysis

#### 3.3.1. General Drosophila Media

**Figure 4:**
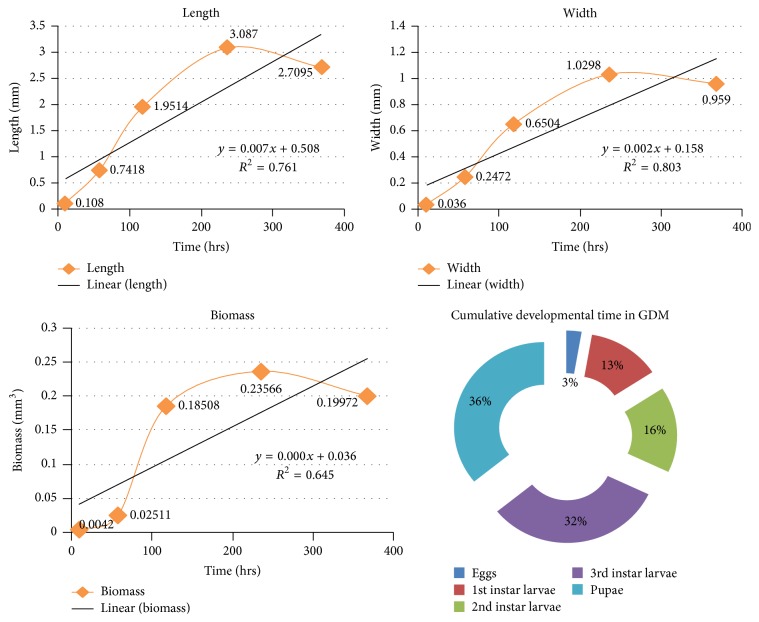


Once the eggs hatched (mean length = 0.1080 ± 0.00261 mm), the first instars larvae (mean length = 0.7418 ± 0.06347 mm) started feeding on the media and grew rapidly to the second instars (mean length = 1.9514 ± 0.18684 mm) and ultimately grew to the third instars (mean length = 3.0870 ± 0.0 mm). However after the feeding phase was over, the maggots started to migrate for finding suitable pupation site inside the containers; most of them settled near the top of the container, after finding suitable pupation media; there was a significant decrease of length, once the postfeeding stage started, the pupa (mean length = 2.7905 ± 0.09074 mm) (*p* < 0.05). The duration of all stages of larvae was also significantly different (*p* < 0.05) (see Table 1 and Figure 4).

#### 3.3.2. Enhanced Drosophila Medium

**Figure 5:**
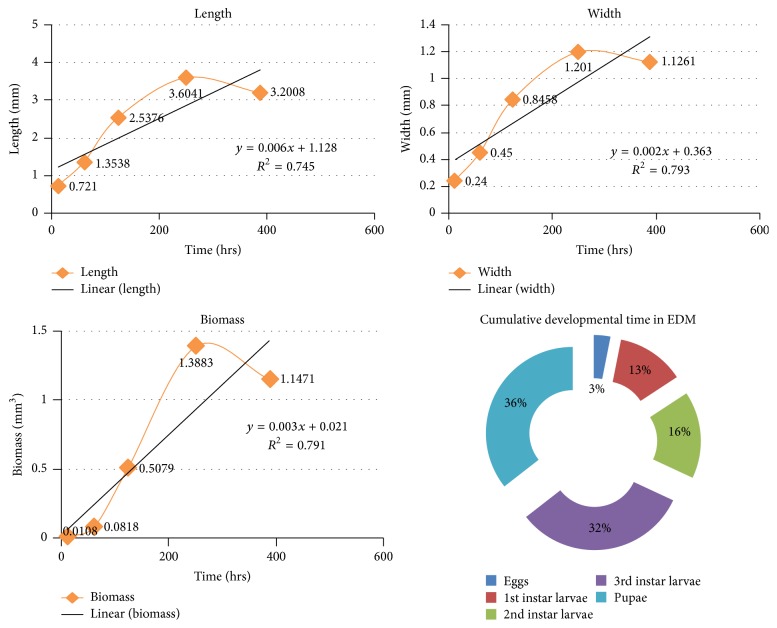


Once the eggs hatched (mean length = 0.7210 ± 0.00260 mm), the first instars larvae (mean length = 1.3538 ± 0.14193 mm) started feeding on the media and grew rapidly to the second instars (mean length = 2.5376 ± 0.17300 mm) and ultimately grew to the third instars (mean length = 3.6041 ± 0.09835 mm). However after the feeding phase was over, the maggots started to migrate for finding suitable pupation site inside the containers; most of them settled near the top of the container, after finding suitable pupation media; there was a significant decrease of length, once the postfeeding stage started, the pupa (mean length = 3.2008 ± 0.09705 mm) (*p* < 0.05). The duration of all stages of larvae was also significantly different (*p* < 0.05) (see Table 2 and Figure 5).

#### 3.3.3. Soy Milk SiO_2_ Media

**Figure 6:**
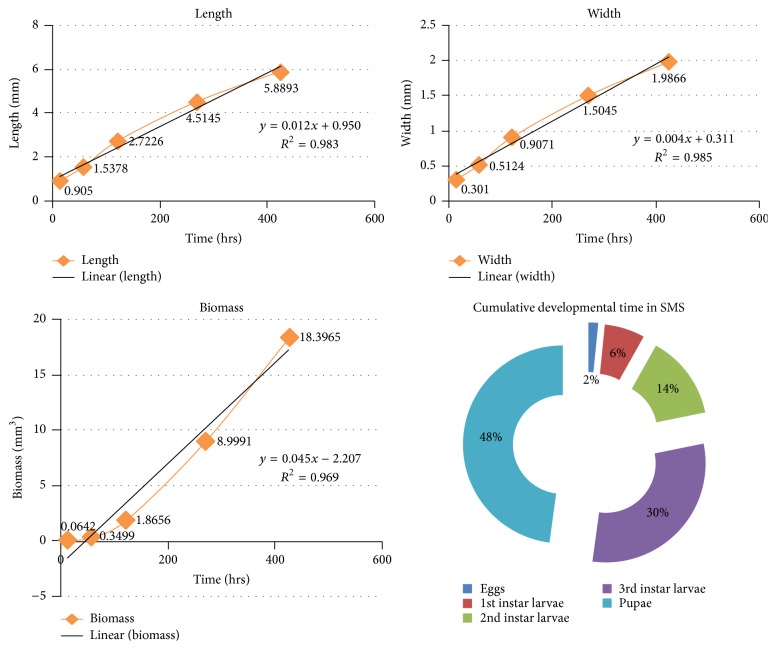


Once the eggs hatched (mean length = 0.9050 ± 0.00260 mm), the first instars larvae (mean length = 1.5378 ± 0.14193 mm) started feeding on the media and grew rapidly to the second instars (mean length = 2.7226 ± 0.17353 mm) and ultimately grew to the third instars (mean length = 4.5145 ± 0.30010 mm). However after the feeding phase was over, the maggots started to migrate for finding suitable pupation site inside the containers; most of them settled near the top of the container, after finding suitable pupation media; there was a significant increase of length, once the postfeeding stage started, namely, the pupa (mean length = 5.8893 ± 0.08514 mm). The duration of all stages of larvae was also significantly different (*p* < 0.05) (see Table 3 and Figure 6).

## 4. Conclusion

**Figure 7:**
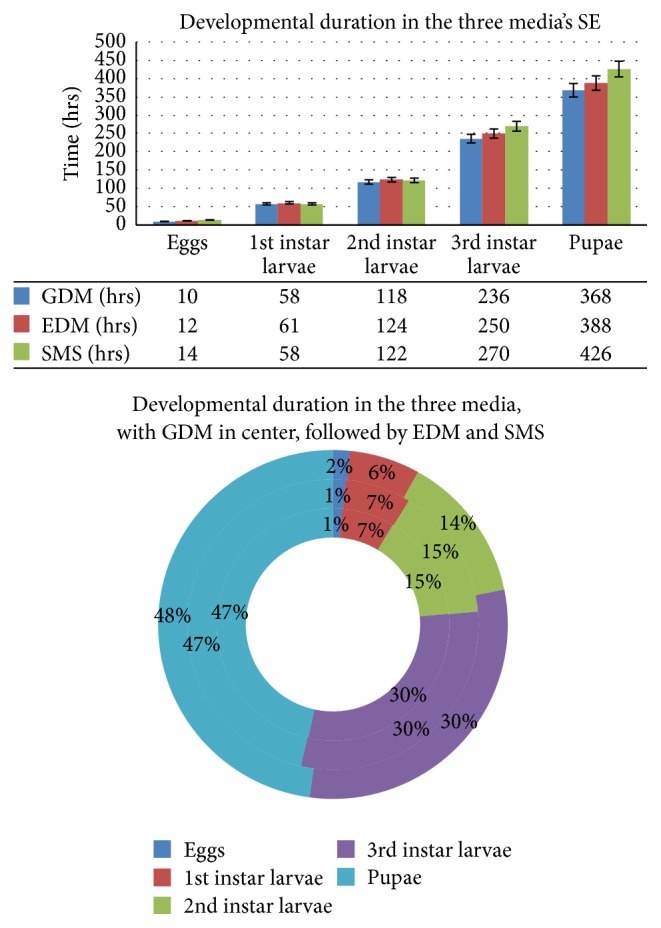


Therefore after analyzing the results obtained from all the three media using ANOVA (1-way) and then by post hoc *t*-test (Bonferroni corrected) (Figure 7), it is found that the SMS medium is a better growth medium for the* Megaselia (M.) scalaris*, in terms of length, width, and biomass of its various developmental stages. The present study provides baseline data on the growth and developmental pattern of the* Megaselia (M.) scalaris*, which, when used in combination with specific geoclimatic reference data, can be applied for forensic entomological studies and also in the usage of* Megaselia (M.) scalaris* as a biocontrol agent of some pestiferous insects.

## Figures and Tables

**Table 1:** 

Stages	Avg. hours (mean ± SE)	Cumulative avg. days	Avg. length (in mm) (mean ± SE)	Avg. width (in mm) (mean ± SE)	Avg. biomass (in mm^3^) (mean ± SE)
Eggs	10 ± 2	10 hrs	0.1080 ± 0.00261	0.0360 ± 0.00870	0.0000 ± 0.00420
1st instar larvae	48 ± 3	2 days, 10 hrs	0.7418 ± 0.06347	0.2472 ± 0.04711	0.0515 ± 0.02511
2nd instar larvae	60 ± 2	4 days, 22 hrs	1.9514 ± 0.18684	0.6504 ± 0.06224	0.7356 ± 0.18508
3rd instar larvae	118 ± 2	9 days, 20 hrs	3.0870 ± 0.09394	1.0298 ± 0.03123	2.6355 ± 0.23566
Pupae	132 ± 4	15 days, 8 hrs	2.7095 ± 0.09074	0.9590 ± 0.02441	2.0204 ± 0.19972

**Table 2:** 

Stages	Avg. hours (mean ± SE)	Cumulative avg. days	Avg. length (in mm) (mean ± SE)	Avg. width (in mm) (mean ± SE)	Avg. biomass (in mm^3^) (mean ± SE)
Eggs	12 ± 2	12 hrs	0.7210 ± 0.00260	0.2400 ± 0.00870	0.0108 ± 0.00166
1st instar larvae	49 ± 2	2 days, 13 hrs	1.3538 ± 0.14193	0.4514 ± 0.04718	0.0818 ± 0.02472
2nd instar larvae	63 ± 3	5 days, 4 hrs	2.5376 ± 0.17300	0.8458 ± 0.05765	0.5079 ± 0.09364
3rd instar larvae	126 ± 3	10 days, 10 hrs	3.6041 ± 0.09835	1.2010 ± 0.03277	1.3883 ± 0.11401
Pupae	138 ± 4	16 days, 4 hrs	3.2008 ± 0.09705	1.1261 ± 0.02635	1.1471 ± 0.09194

**Table 3:** 

Stages	Avg. hours (mean ± SE)	Cumulative avg. days	Avg. length (in mm) (mean ± SE)	Avg. width (in mm) (mean ± SE)	Avg. biomass (in mm^3^) (mean ± SE)
Eggs	14 ± 1	14 hrs	0.9050 ± 0.00260	0.3010 ± 0.00870	0.0642 ± 0.00734
1st instar larvae	44 ± 2	2 days, 10 hrs	1.5378 ± 0.14193	0.5124 ± 0.04740	0.3499 ± 0.09483
2nd instar larvae	64 ± 2	5 days, 2 hrs	2.7226 ± 0.17353	0.9071 ± 0.05783	1.8656 ± 0.32497
3rd instar larvae	148 ± 1	11 days, 6 hrs	4.5145 ± 0.30010	1.5045 ± 0.10002	8.9991 ± 1.72950
Pupae	156 ± 2	17 days, 18 hrs	5.8893 ± 0.08514	1.9866 ± 0.02574	18.3965 ± 0.82974
